# Characteristics of Three Forensic Veterinary Cases Involving Pet Deaths in Domestic Murder-Suicide Incidents

**DOI:** 10.3390/ani15172504

**Published:** 2025-08-26

**Authors:** Yuko Kihara, Yohsuke Makino, Suguru Torimitsu, Fumiko Chiba, Hirotaro Iwase, Makoto Nakajima, Aki Tanaka

**Affiliations:** 1 Laboratory of Shelter Medicine, Faculty of Veterinary Science, Nippon Veterinary and Life Science University, 1-7-1 Kyonan-cho, Tokyo 180-8602, Japan; atanaka@nvlu.ac.jp; 2 Department of Forensic Medicine, Graduate School of Medicine, The University of Tokyo, 7-3-1 Hongo, Tokyo 113-0033, Japan; ymakino@m.u-tokyo.ac.jp (Y.M.); torimitsu@m.u-tokyo.ac.jp (S.T.); chibafumico@chiba-u.jp (F.C.); iwase@faculty.chiba-u.jp (H.I.); makoto@m.u-tokyo.ac.jp (M.N.); 3 Education and Research Center of Legal Medicine, Graduate School of Medicine, Chiba University, 1-8-1 Inohana, Chiba 260-8670, Japan; 4 One Health and One Welfare Research Center, Faculty of Veterinary Science, Nippon Veterinary and Life Science University, 1-7-1 Kyonan-cho, Tokyo 180-8602, Japan

**Keywords:** murder-suicide-peticide, family violence, forensic necropsy, one welfare, animal abuse

## Abstract

Murder-suicide, also referred to as homicide-suicide, results in multiple casualties. The victims are typically human, and cases involving the inclusion of pets, sometimes termed “murder-suicide-peticide”, have been rarely reported worldwide. The aim of this study is to present and examine the characteristics of three forensic veterinary cases in which pets were found dead alongside human victims in suspected domestic murder-suicide incidents in Japan. We analyzed information provided by the police agency about the owners, families, and the animals, as well as necropsy findings of three cases. In all three cases, it was determined that both the human and animal victims died from the same cause, suggesting a tendency for perpetrators to kill pets using the same methods as those used on human victims. Murder-suicide is regarded as the most extreme form of domestic violence and abuse. Ongoing interdisciplinary research is essential for developing effective intervention strategies to protect vulnerable individuals, both human and non-human, within the household.

## Simple Summary

This study presents and examines the characteristics of three forensic veterinary cases in which pets were found dead alongside human victims in suspected domestic murder-suicide incidents. In all three cases, it was suggested that pets are often regarded by perpetrators as members of the family and, as such, may become victims of murder-suicide alongside human household members.

## 1. Introduction

Murder-suicide (also referred to as homicide-suicide) is a phenomenon in which an individual kills others and then attempts to take their own life. It often results in multiple casualties. Such incidents may also include cases referred to as “Muri-shinju”, often within familial or close relationships in Japan. A study of 17 countries [1], including Japan, reported that the murder-suicide rate per 100,000 people ranged from 0.012 in Scotland to 0.13 in Greenland, which accounted for approximately 8% of all murders. The relationship between the victim and the perpetrator in a murder-suicide is not limited to relatives, but also includes cases where unrelated strangers are killed or injured. On the other hand, in domestic homicide-suicide, the perpetrator kills their intimate partner and/or children and themselves. Victims are typically human, and this phenomenon is regarded as an extreme form of domestic violence [2]. Reports of cases involving pets—sometimes termed “murder-suicide-peticide”—remain scarce worldwide [3,4], so little is known about cases that involve pets.

The concept of One Welfare has been gaining recognition in the field of veterinary medicine in the past decade [5,6,7]. This framework emphasizes the interconnectedness of human well-being, animal welfare, and the sustainability of society and the environment. It complements the One Health approach, which focuses on the interrelationship between the health of animals, humans, and the global environment. Animal welfare is an academic field that scientifically evaluates the condition of animals kept under human care, considering how well their physical and psychological needs are being met, including their living environments. In promoting One Welfare, veterinarians are expected to contribute to improvements in human health, well-being, public health, and environmental hygiene by advancing animal welfare.

The present study aims to contribute to the promotion of One Welfare by presenting and analyzing three cases of animal necropsies conducted by veterinarians in suspected domestic murder-suicide incidents involving the deaths of both human and animal victims, including the completed or attempted suicide cases.

## 2. Method

We collected and analyzed three murder-suicide-peticide cases in Japan. In all three cases, the animal bodies were examined through forensic necropsy at the authors’ veterinary university. Postmortem CT was conducted before necropsy. However, histopathological examinations were not performed in all animal cases, since prompt necropsy reports were required. Among the human victims, only Case 2 underwent forensic autopsy at the authors’ department of forensic medicine; the other two autopsies were conducted at external institutions. Accordingly, information regarding the human deaths in Cases 1 and 3 was obtained from police reports and publicly available news sources.

## 3. Case Presentation

### 3.1. Case 1

Province, year, and season: Case 1 occurred in the Kyushu district during the summer of 2020.

#### 3.1.1. Owner and Pet

**Figure 1:**
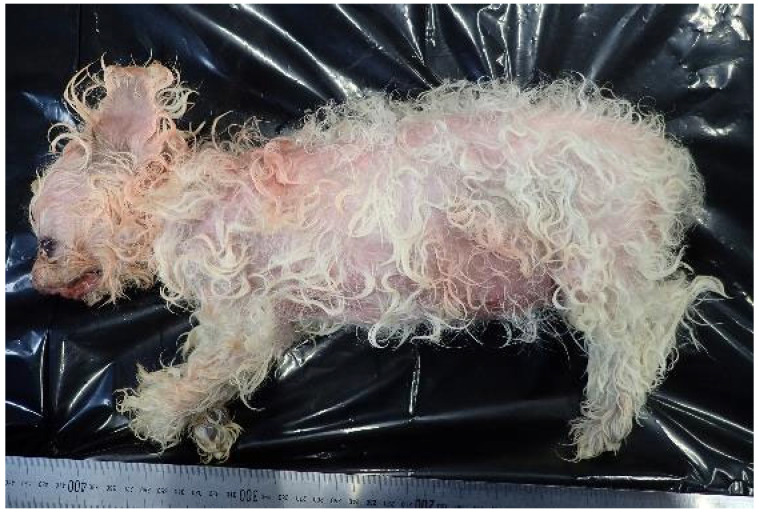
The left side of the whole body of Case 1.

A couple in their 70s were living with a 16-year-old female mixed-breed dog (Maltese and Chihuahua mix) (Figure 1).

#### 3.1.2. Circumstances at Discovery

The incident was reported by the couple’s son, who lived on the same property and called emergency services. The husband was found and transported to a hospital, where he was pronounced dead. The suspect, the wife, was found unconscious in the home’s bathtub, having attempted suicide by submerging herself in water. The dog was found dead in her arms.

#### 3.1.3. Cause of Death—Human Victim (Husband)

According to a press release from the local police department, the cause of death was asphyxiation due to neck compression. The victim was physically disabled and had difficulty walking, suffered from dementia, and was being cared for by the perpetrator. There were no signs of resistance against the perpetrator on his body.

#### 3.1.4. Cause of Death—Dog

**Figure 2:**
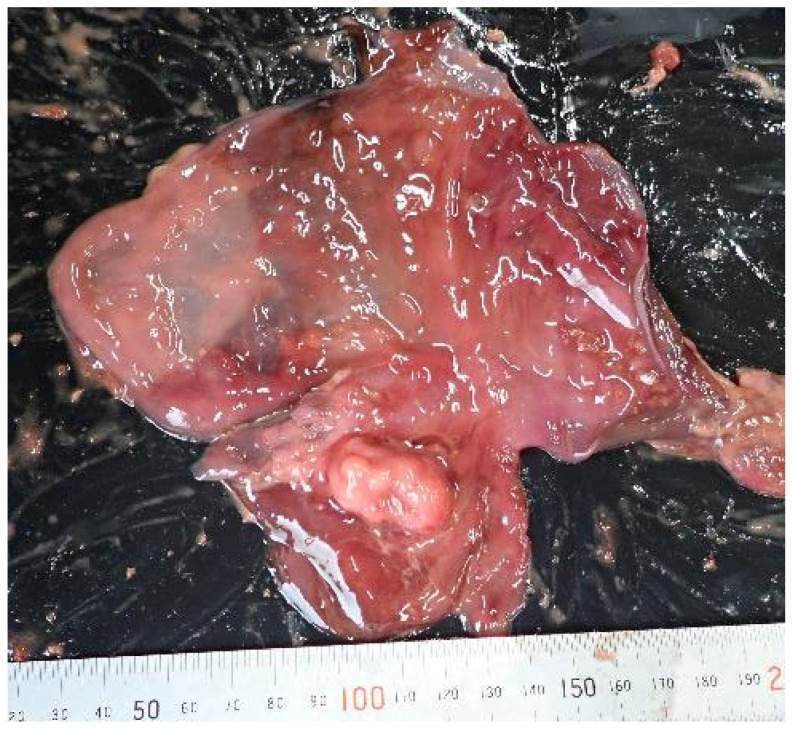
A 2.5 cm × 1.5 cm mass on the gastric mucosa of Case 1.

**Figure 3:**
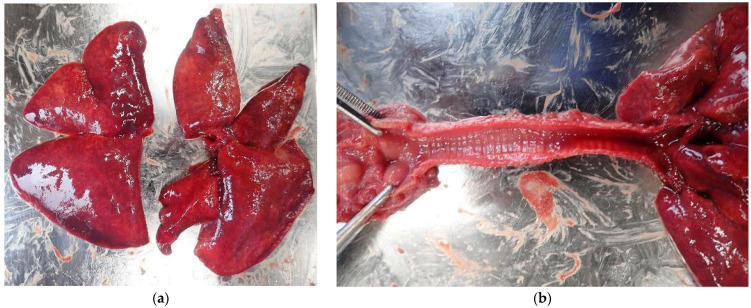
The lung and the trachea of Case 1: (**a**) gross appearance of the lungs show congestion; (**b**) no fluid accumulation in the trachea.

**Figure 4:**
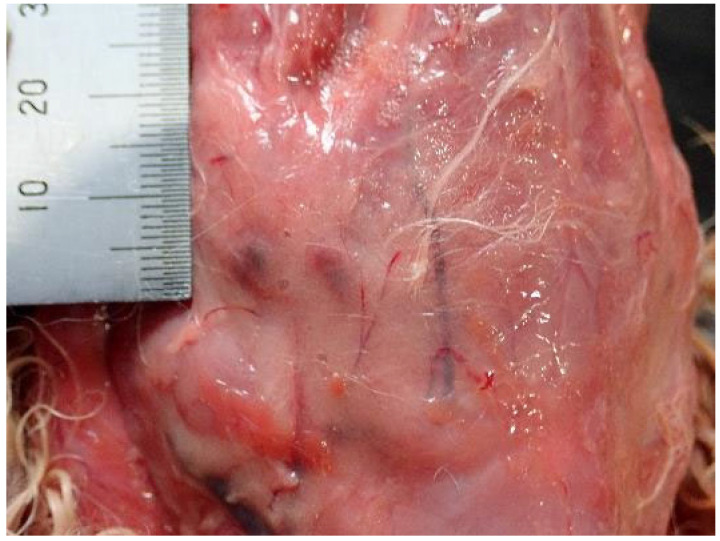
Subcutaneous hemorrhages observed on right side of the mandibular angles of Case 1.

A 2.5 cm × 1.5 cm mass was observed on the gastric mucosa; however, this finding was not considered fatal (Figure 2). Typical signs of drowning, such as pulmonary edema and fluid accumulation in the trachea, were absent (Figure 3). However, subcutaneous hemorrhages were observed on both sides of the mandibular angles (Figure 4), and the cause of death was determined to be asphyxiation due to neck compression.

#### 3.1.5. Suspected Motive (Based on Police Information)

The suspect stated that she killed her husband due to exhaustion from caregiving responsibilities. No information was obtained regarding the motive for killing the dog.

### 3.2. Case 2

Province, year, and season: Case 2 occurred in the Kanto district during the summer of 2022.

#### 3.2.1. Owner and Pet

**Figure 5:**
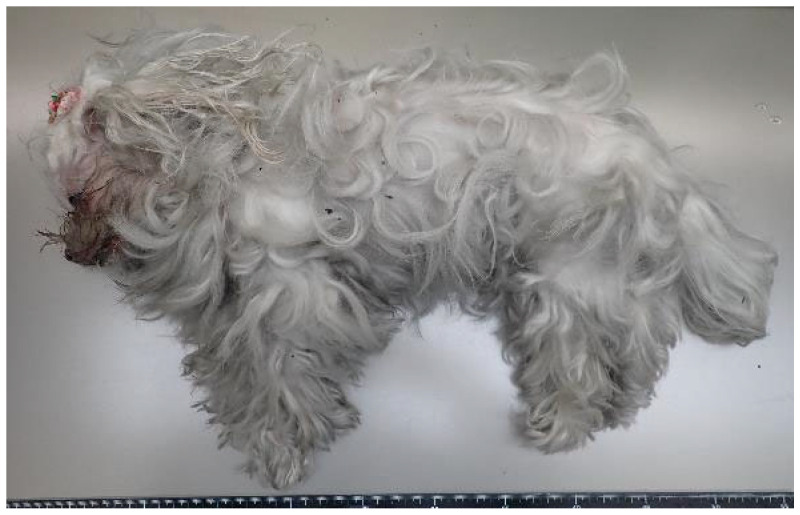
The left side of the whole body of Case 2.

A common-law couple consisting of a man in his 70s and a woman in her 50s kept a 7-year-old female Maltese dog (Figure 5).

#### 3.2.2. Circumstances at Discovery

The incident was reported by a passerby who witnessed a house fire and called emergency services. The man was found collapsed naked in the first-floor bathroom, and the woman was found lying on a Japanese futon on the second floor. Both were transported to a hospital, where they were pronounced dead. There was video footage of the perpetrator purchasing kerosene at a gas station. The domestic oil tank was placed in the hallway in front of the bathroom, and signs of oil spillage were detected in this area, which led the police to estimate that this was the location of the fire. The following day, during a joint on-site investigation by the fire department and police, the dog was found dead on a sofa in the first-floor living room, under a blanket.

#### 3.2.3. Cause of Death—Perpetrator (Female)

A forensic autopsy revealed approximately 12% of the body surface had sustained second- to third-degree burns. Thermal injuries to the airway, soot deposition in the trachea, and hypoxic encephalopathy were observed. The cause of death was determined to be carbon monoxide (CO) poisoning related to the fire. Other findings were revealed, including enlarged ovaries on both sides, enlarged lymph nodes in the left pelvic wall, numerous nodules in the peritoneum, and multiple masses in the lungs and liver. These findings indicated terminal ovarian cancer with peritoneal dissemination and metastasis to the lymph nodes, lungs, and liver. However, she had no history of medical consultations.

#### 3.2.4. Cause of Death—Victim (Male)

A forensic autopsy revealed approximately 30% of the body surface had erythema, and soot was observed in the airway. The cause of death was determined to be death by fire.

#### 3.2.5. Cause of Death—Dog

**Figure 6:**
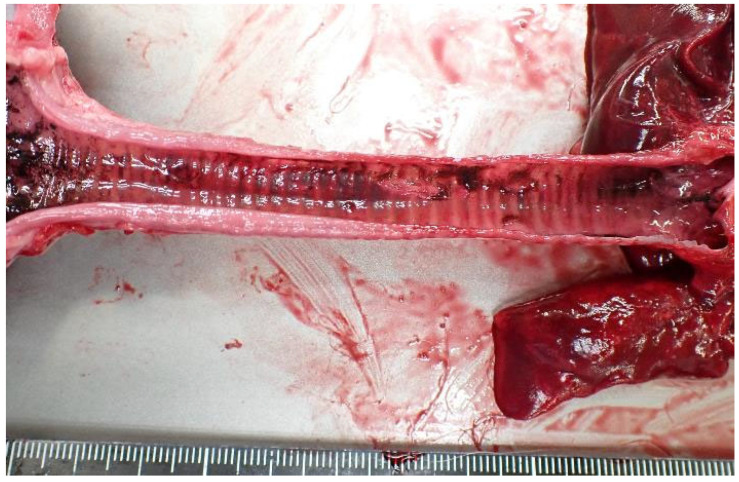
Soot in the trachea.

**Figure 7:**
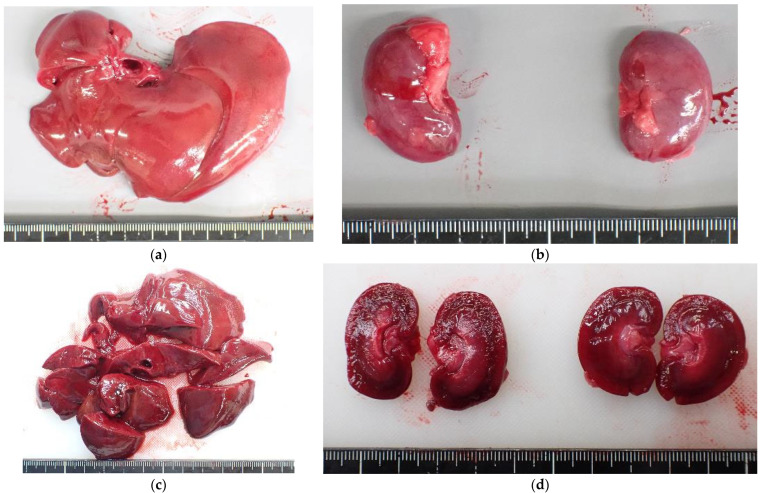
Bright red discoloration: (**a**) in surface of the liver; (**b**) in the kidney; (**c**) in the cut surface of the liver; (**d**) in the kidney.

Soot was present across the body and in the trachea (Figure 5 and Figure 6). Bright red discoloration was observed in the heart blood, visceral surfaces (Figure 7), and muscle tissue. CO-hemoglobin (CO-Hb) levels exceeded the detection limit at 75% in both the left and right sides of the heart, and the cause of death was diagnosed as CO poisoning associated with the fire. CO-Hb levels were measured using a blood gas analyzer (AVOXimeter 4000, International Technidyne Corp, Edison, NJ, USA).

#### 3.2.6. Suspected Motive (Based on Police Information)

No suicide note or statements from relatives were available, and the motive remains unknown. In addition, no evidence was found to determine that this case was a murder-suicide. However, while there is no conclusive evidence that the perpetrator started the fire, suffering of sickness is suspected as the perpetrator’s motive for the murder-suicide. It is likely that the perpetrator had set fire to the hallway in front of the bathroom while the victim was bathing, resulting in the deaths of both victims due to inhalation of CO from the fire.

### 3.3. Case 3

Province, year, and season: Case 3 occurred in the Kanto district during the winter of 2021.

#### 3.3.1. Owner and Pet

**Figure 8:**
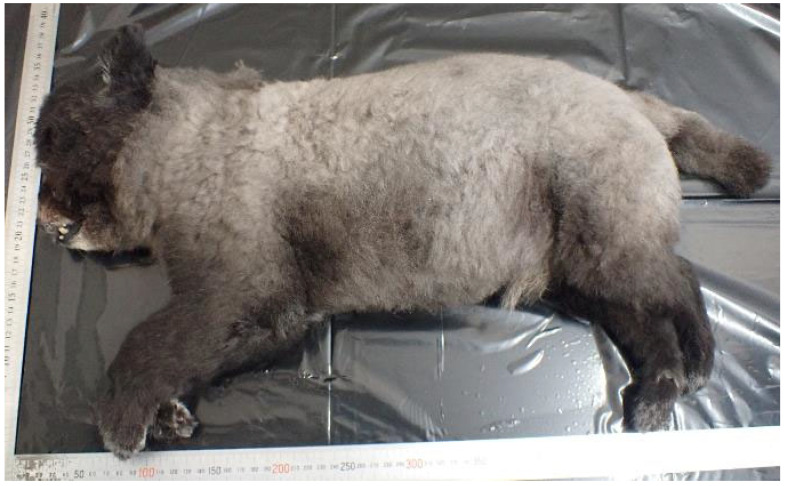
The left side of the whole body of Case 3.

A woman in her 40s, living with her father in his 70s, owned one 8-year-old male Pomeranian dog (Figure 8).

#### 3.3.2. Circumstances at Discovery

Police searched the residence after receiving a request from relatives for a welfare check. The father and daughter were found deceased inside the home. The dog was found dead inside the father’s clothing. Based on the police investigation, it was estimated that approximately two weeks had passed since death.

#### 3.3.3. Cause of Death—Victim (Daughter)

According to police reports, there was sign of strangulation, as if something had been wrapped around her neck. She was presumed to have died from asphyxiation due to neck compression.

#### 3.3.4. Cause of Death—Perpetrator (Father)

The father was found in a seated position with a tie around his neck and with signs consistent with atypical hanging. He was presumed to have died from asphyxiation due to neck compression.

#### 3.3.5. Cause of Death—Dog

**Figure 9:**
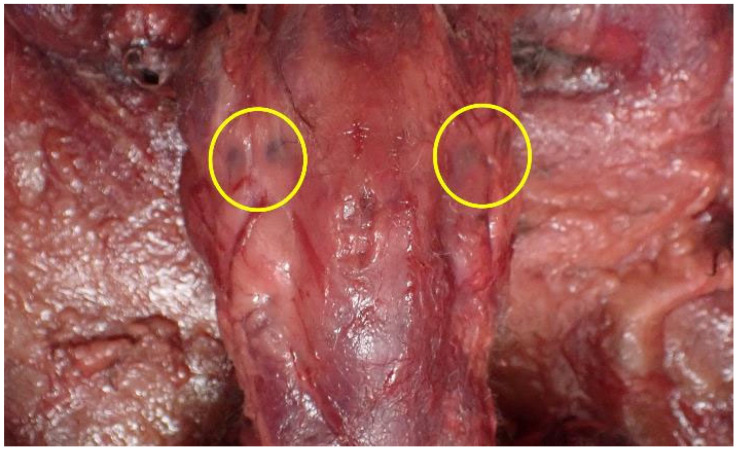
Subcutaneous hemorrhages observed on both sides of the mandibular angles of Case 3. (yellow circles).

**Figure 10:**
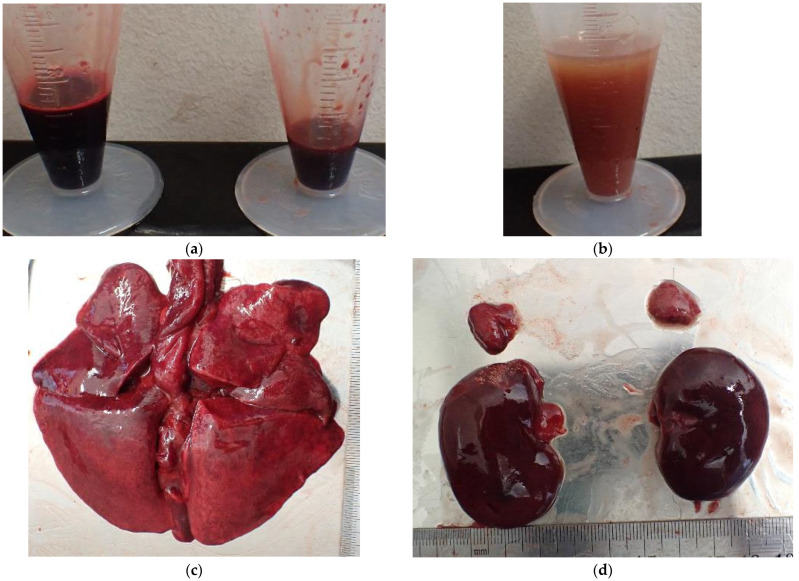
Necropsy findings of Case 3: (**a**) fluid accumulation in the thoracic; (**b**) abdominal cavities; (**c**) congestion of the lungs; (**d**) congestion of the kidney.

Subcutaneous hemorrhages were observed on both sides of the mandibular angles (Figure 9). Fluid accumulation was noted in the thoracic and abdominal cavities, along with congestion of internal organs (Figure 10). Based on these findings, the cause of death was determined to be asphyxiation due to neck compression.

#### 3.3.6. Suspected Motive (Based on Police Information)

According to statements from relatives, frequent arguments between the father and daughter had been reported. No information was obtained regarding the motive for killing the dog.

The characteristics of the above three cases are summarized in Table 1.

## 4. Discussion

This study is the first to examine forensic veterinary necropsies of pet animals and the first in Japan to collect and analyze three murder-suicide cases in which companion animals were also killed. The findings suggest that, in Japan as in other countries, pets are sometimes treated as members of the family and may be included as targets in murder-suicide incidents [3,4].

None of the media articles covering these three cases mentioned the killing of the dogs. Given that murder-suicide cases involving humans often attract considerable media attention [8] and are frequently reported in Japan, the lack of public awareness regarding murder-suicide-peticide incidents may reflect a broader social invisibility of such events. This underreporting could be one of the reasons why preventive measures against murder-suicide-peticide remain underdeveloped. Case analyses such as the present study, which collect and document such rare incidents, provide valuable information for future interdisciplinary research and policy consideration.

In terms of age, both the perpetrator and the victim were in their 70s in two of the three cases. Regarding sex, the perpetrators were female in two cases, while the victims were male in two cases. As for the relationship between the individuals involved, two cases involved spouses or common-law partners, and one case involved a parent–adult child relationship.

Previous studies on murder-suicide have reported that both perpetrators and victims are often aged 60 years or older [1,9]. In terms of sex, male perpetrators and female victims are more common [1,4,9,10,11]. As for the nature of the relationship, spousal or common-law partnerships are the most frequently reported in many countries [1,10,11], followed by parent–child and sibling relationships [1]. In contrast, studies in Japan have suggested that murder-suicide cases more commonly occur between adult children and their parents [9].

In a previous study from Japan [9], perpetrators aged >65 years comprised 29/77 (38%) cases, female perpetrators comprised 25/77 (33%) cases, victims aged >65 years comprised 38/89 (43%) cases, and female victims comprised 64/89 (72%) cases. Relationships were older parent–adult child in 19/77 (25%), parent–child in 17/77 (22%), and older spouses in 16/77 (20%) cases [9]. The three cases examined in the present study are generally consistent with trends reported in Japan in terms of age and relationship—most of the victims and perpetrators in this study were aged 60 years or older, and spousal or common-law partnerships made up two cases, followed by a parent–child relationship in one case. Regarding sex, male victims were more common in this sample. Notably, one of the male victims had been receiving caregiving support from the female perpetrator, suggesting that he may have been a vulnerable member within the household.

Regarding animal species, all three cases involved small toy dog breeds: two Maltese or Maltese-mix dogs and one Pomeranian. Each case involved a single dog, and all were middle-aged or older. Previous studies have also reported that dogs are the most commonly involved species in such cases, followed by cats and rabbits [4], and that the most frequent number of animal victims per case is one [4]. According to national estimates based on feeding volumes by the Japan Pet Food Association, 2024, the number of pet cats in Japan is approximately 9.16 million, while that of pet dogs is around 6.80 million—indicating that cats are more commonly kept than dogs [12]. Nevertheless, dogs appear more frequently as victims in murder-suicide-peticide cases. One possible explanation is that cats, being more adept at escaping and hiding, may be less likely to be caught up in such incidents [4]. Therefore, the involvement of dogs may represent a characteristic feature of murder-suicide-peticide cases in Japan.

A previous study on pet attachment in Japan found that female owners tend to report stronger emotional bonds with their pets than male owners, and that dog owners are more attached to their pets than cat owners [13]. In the present study, two of the three perpetrators were female dog owners, which may suggest that not only close human companions but also deeply bonded pets were intentionally included in the acts of violence.

All three incidents took place within the family’s home. In murder-suicide cases, the perpetrator often displays a strong intention to complete the act without interference. This may explain why such incidents frequently occur in private residences, where there is a minimal risk of the victim escaping or of third-party intervention. In Case 3, the bodies were not discovered until approximately two weeks after death, suggesting the family may have been socially isolated. Although research on murder-suicide remains limited and effective intervention strategies have yet to be established, previous studies have similarly noted that these incidents often occur within the home [1,4]. Considering this tendency, it is important to prevent the psychological and physical isolation of socially vulnerable individuals, such as those burdened by caregiving responsibilities, chronic illness, or financial hardship.

Regarding cause of death, two of the perpetrators died by suicide—one by asphyxiation due to neck compression and one by CO poisoning—while one case involved a failed suicide attempt by drowning. Among the human victims, two died from asphyxiation due to neck compression, and one died from fire-related injuries. The animal victims died from asphyxiation in two cases and from CO poisoning in one case. Although in many other countries domestic homicides most often involve firearm injuries [4,10,11], in Japan neck compression is most common [9]. This discrepancy likely reflects Japan’s strict gun control laws. Hong Kong also has strict firearm regulations, and reports of suffocation, sharp instrument injuries, and CO poisoning as causes of death in homicide-suicide cases have been reported [1,14]. Taken together, these findings suggest that asphyxiation by neck compression may be a predominant cause of death in murder-suicide-peticide cases in Japan, which has strict firearm regulations. Moreover, neck compression may be easy for the perpetrators, as the animals are relatively small in Japanese households [12].

In Case 2, the perpetrator and the pet died from CO poisoning, while the human victim died from fire-related injuries; however, all deaths were caused by the same fire and are thus classified as fire-related. Overall, in all three cases, the causes of death for the human and animal victims were the same. This indicates a tendency for both people and pets to be killed by the same method in murder-suicide-peticide incidents.

Regarding motive, although only presumptive information was available from police sources, caregiver fatigue was suspected in one case, family-related stress in another, and the motive remained unknown in the third. In all three cases, the motive for killing the pet was not identified. Previous studies have indicated that suicidal ideation [1], interpersonal stress (especially involving family members) in more than half of the cases [11], as well as the perpetrator’s physical illness, financial hardship, morbid jealousy, and postpartum depression [1] have all been cited as potential motives for murder-suicide.

In studies involving pets as part of murder-suicide incidents, it has been suggested that, in addition to interpersonal stress and health-related issues, some perpetrators may engage in so-called “mercy killings” of pets due to concerns that no one would be available to care for them after the perpetrator’s death [4]. However, whether the animal death was in connection to the murder-suicide or not, killing pet animals is prohibited in Japan, as in many countries. In Cases 1 and 3, necropsy revealed compression of the dog’s lower jaw, which constituted evidence of violence, clearly indicating animal abuse. In Case 2, a dog died of CO poisoning caused by a fire inside a home, with no route for the dog to escape the fire. This dog’s body had been found under a blanket on a sofa; this suggests that the dog may have attempted to escape the fire. Leaving a pet dog in a dangerous indoor fire situation is animal abuse. Therefore, all three cases were not only murder-suicide, but also animal abuse, causing the death of the animals by violence and neglect without considering giving the pets away, rehoming them, or putting them outside of the home to be found by someone.

The present study is based on animal forensic necropsy cases, in which information relevant to the animals’ causes of death was disclosed by police to the attending veterinarians. However, detailed information regarding the human victims’ causes of death, history of domestic violence, previous incidents of animal abuse or neglect, veterinary treatment history, and whether legal proceedings were initiated concerning the killing of the animal after death were not provided. In Japan, investigative information is typically retained within police departments and not shared externally. As a result, conducting epidemiological studies to explore background factors related to criminal cases or accidents remains difficult [15], which represents the limitation of this study.

In Case 2, toxicological analysis of human autopsy specimens revealed no detectable levels of ethanol or psychotropic drugs. In murder-suicide cases, psychotropic agents such as sleeping pills or benzodiazepines are sometimes detected in the bodies of perpetrators or victims, particularly in children [9,14]. This highlights the importance of conducting toxicological examinations on animal remains in murder-suicide-peticide cases as well. However, in the present three cases, no toxicological testing targeting psychotropic substances was performed on the animals. Although one of the barriers to such testing in animals is the high financial cost associated with comprehensive toxicological screening, it is recommended that toxicological testing for psychotropic drugs in the animal cases is conducted in the future, as in human cases.

**Figure 11:**
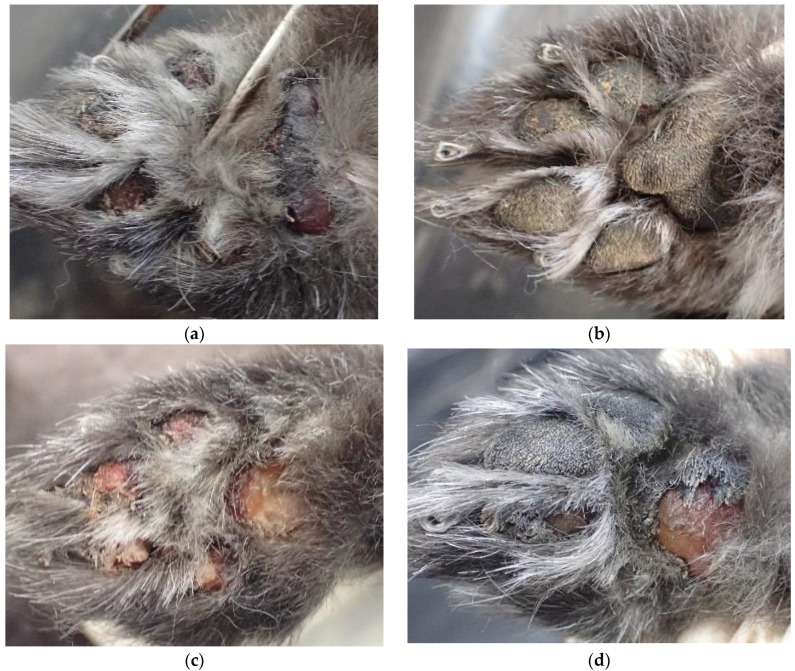
Necropsy findings of Case 3, indicating prior abuse: (**a**) epidermal detachment of the digital and metacarpal pads of the right forelimb; (**b**) no lesion on the left forelimb; (**c**) the right hindlimb; (**d**) the left hindlimb.

In the dog from Case 3, in addition to findings consistent with asphyxiation due to neck compression, forensic necropsy revealed epidermal detachment of the digital and metacarpal pads (excluding the left forelimb) (Figure 11) and subcutaneous hemorrhage on the dorsal surface. Notably, the absence of bleeding in the detached pads suggested that these were chronic injuries, indicative of prior abuse. Therefore, in Case 3, it is possible that signs of animal abuse could have served as an early indicator of problems within the household prior to the murder-suicide event. This underscores the role of veterinarians not only in assessing the health of animals to detect animal abuse, but also in identifying potential issues in the home environment where the animals are kept.

The present murder-suicide-peticide cases reveal a constant pattern in which socially and psychologically isolated individuals kill close human companions and emotionally bonded pets using the same method, typically within the privacy of their homes where external intervention is unlikely. In particular, Case 2 presented findings suggestive of prior animal abuse, indicating the potential for early identification of household problems through veterinary examination before a murder-suicide occurs. This highlights the importance of information sharing and interdisciplinary collaboration among professionals in veterinary medicine, human medicine, social services, and law enforcement. The absence of media coverage on murder-suicide-peticide cases may contribute to the lack of public awareness and delayed policy response in Japan. Given that these incidents severely compromise the welfare of both humans and animals, it is essential to continue documenting such cases. Promoting transdisciplinary research across fields such as veterinary medicine, human medicine, and public health, and disseminating research findings widely will be crucial in advancing understanding and prevention of these complex events.

## 5. Conclusions

Pets are often regarded by perpetrators as members of the family and, as such, may become victims of murder-suicide alongside human household members. Murder-suicide is considered the most extreme form of domestic violence and abuse. To develop effective intervention strategies for protecting vulnerable individuals within the household, research that transcends disciplinary boundaries encompassing medicine, veterinary medicine, public health, and social welfare is essential.

## Figures and Tables

**Table 1:** Characteristics of three forensic veterinary cases involving pet deaths in murder-suicide incidents within households in 2020–2022. The information in Table 1 comes from the analysis of the three cases.

	Perpetrator (*N*)	Human Victims (*N*)	Animal Victims (*N*)
Age	50s (1), 70s (2)	40s (1), 70s (2)	Middle age (3)
Sex	Male (1), Female (2)	Male (2), Female (1)	Male (1), Female (2)
Animal species and numbers	-	-	One toy dog (3)
The site of death	Bedroom (1), Unknown (1)	Bathroom (1), Unknown (2)	Living room (1), Bathroom (1), Inside shirt (1)
The cause of death	Completed suicide (2): Asphyxiation due to neck compression (1), Carbon monoxide poisoning (1); Attempted suicide (1): Drowning (1)	Asphyxiation due to neck compression (2), Death by fire (1)	Asphyxiation due to neck compression (2), Carbon monoxide poisoning (1)
Motive	Exhaustion from caregiving responsibilities (1), Family-related stress (1), Unknown (1)		

## Data Availability

This article contains all data found in this study. Further details or information can be directed to the corresponding author.
